# Supplementary material for: Non-iterative Triples for Transcorrelated Coupled Cluster Theory
Source: J Chem Theory Comput. 2025 Feb 17;21(4):1588–601. doi: 10.1021/acs.jctc.4c01062 (PMC11866761; doi:10.1021/acs.jctc.4c01062)
Supplement: Supplementary file 1 — ct4c01062_si_001.pdf [file ct4c01062_si_001.pdf]

Non-iterative triples for transcorrelated coupled cluster theory

Maximilian Mörchen, Alberto Baiardi, Michał Lesiuk, Markus Reiher

25.1.2025

Supporting Information

1 LiH molecule

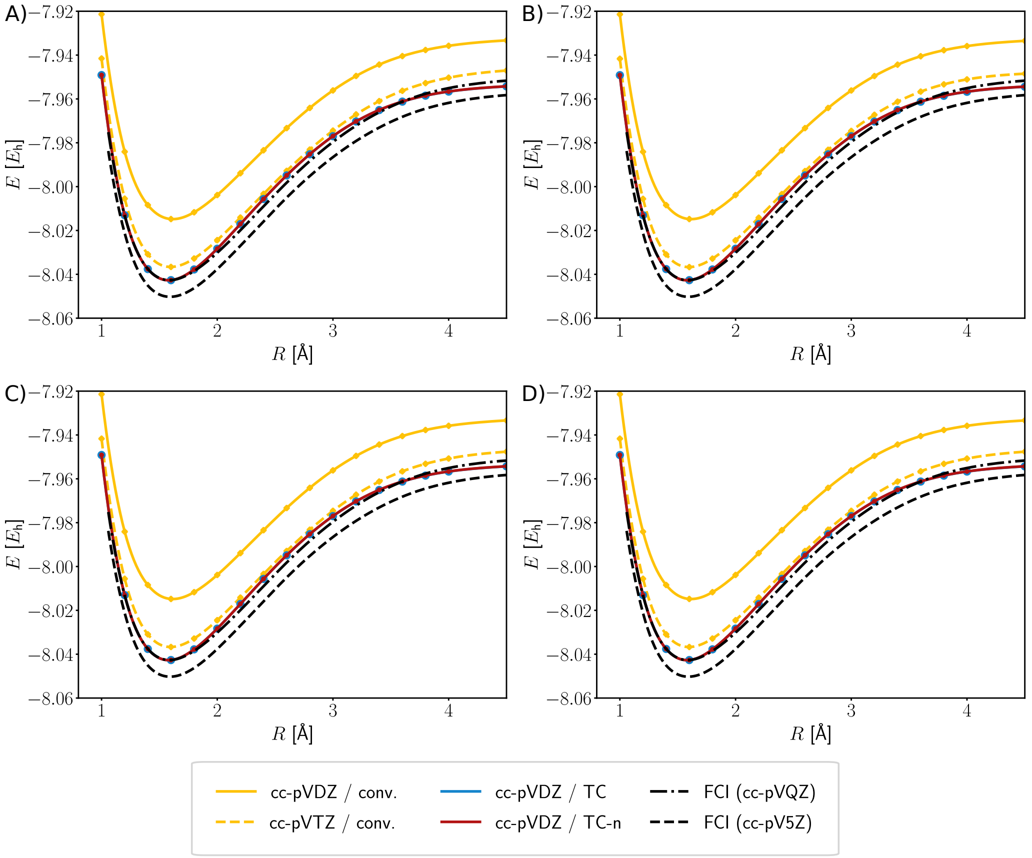

Figure S1: Dissociation of the LiH molecule based on A) CCSD, B) CCSD(T), C) CCSDT and D) CCSDTQ. The absolute energies in Hartree are plotted against the internuclear distance in Å. The Potential energy curves were calculated with the transcorrelated Hamiltonian ('TC' and 'TC-n' for the normal-ordering approximation) and the conventional ('conv.') Hamiltonian in different basis sets. For comparison, we show FCI curves in the cc-pVQZ and cc-pV5Z basis set (taken from Ref. *1*).

	cc-pvdz(conv)	cc-pvtz(conv)	cc-pvdz(TC)	cc-pvdz(TC-n)
1.0	-7.921378	-7.941549	-7.949124	-7.949121
1.2	-7.984086	-8.005483	-8.013033	-8.013029
1.4	-8.008421	-8.030840	-8.037606	-8.037601
1.6	-8.014792	-8.036627	-8.042663	-8.042655
1.8	-8.011697	-8.032779	-8.037784	-8.037775
2.0	-8.003844	-8.024411	-8.028329	-8.028317
2.2	-7.993892	-8.014107	-8.017095	-8.017080
2.4	-7.983412	-8.003307	-8.005657	-8.005638
2.6	-7.973307	-7.992814	-7.994876	-7.994851
2.8	-7.964106	-7.983099	-7.985235	-7.985205
3.0	-7.956122	-7.974451	-7.976995	-7.976957
3.2	-7.949520	-7.967050	-7.970276	-7.970232
3.4	-7.944332	-7.960996	-7.965068	-7.965019
3.6	-7.940455	-7.956281	-7.961226	-7.961174
3.8	-7.937683	-7.952791	-7.958511	-7.958459
4.0	-7.935767	-7.950316	-7.956651	-7.956601
4.5	-7.933285	-7.947041	-7.954275	-7.954231

Table S1: CCSD potential energy curve of the LiH dissociation for different basis sets.

	cc-pvdz(conv)	cc-pvtz(conv)	cc-pvdz(TC)	cc-pvdz(TC-n)
1.0	-7.921425	-7.941675	-7.949151	-7.949148
1.2	-7.984111	-8.005577	-8.013046	-8.013041
1.4	-8.008433	-8.030917	-8.037611	-8.037605
1.6	-8.014801	-8.036695	-8.042665	-8.042658
1.8	-8.011706	-8.032843	-8.037786	-8.037777
2.0	-8.003853	-8.024476	-8.028332	-8.028320
2.2	-7.993902	-8.014175	-8.017098	-8.017083
2.4	-7.983423	-8.003382	-8.005662	-8.005642
2.6	-7.973321	-7.992903	-7.994882	-7.994857
2.8	-7.964124	-7.983208	-7.985244	-7.985213
3.0	-7.956146	-7.974592	-7.977007	-7.976970
3.2	-7.949554	-7.967241	-7.970295	-7.970251
3.4	-7.944381	-7.961265	-7.965098	-7.965049
3.6	-7.940525	-7.956670	-7.961270	-7.961218
3.8	-7.937782	-7.953349	-7.958575	-7.958523
4.0	-7.935898	-7.951092	-7.956739	-7.956688
4.5	-7.933497	-7.948483	-7.954421	-7.954377

Table S2: CCSD(T) potential energy curve of the LiH dissociation for different basis sets.

	cc-pvdz(conv)	cc-pvtz(conv)	cc-pvdz(TC)	cc-pvdz(TC-n)
1.0	-7.921430	-7.941696	-7.949143	-7.949141
1.2	-7.984113	-8.005593	-8.013041	-8.013036
1.4	-8.008435	-8.030931	-8.037609	-8.037603
1.6	-8.014803	-8.036708	-8.042665	-8.042657
1.8	-8.011708	-8.032857	-8.037787	-8.037777
2.0	-8.003855	-8.024490	-8.028333	-8.028321
2.2	-7.993903	-8.014190	-8.017100	-8.017085
2.4	-7.983425	-8.003398	-8.005664	-8.005645
2.6	-7.973322	-7.992919	-7.994886	-7.994860
2.8	-7.964125	-7.983225	-7.985246	-7.985215
3.0	-7.956146	-7.974606	-7.977008	-7.976970
3.2	-7.949549	-7.967244	-7.970293	-7.970249
3.4	-7.944368	-7.961240	-7.965088	-7.965040
3.6	-7.940498	-7.956587	-7.961251	-7.961200
3.8	-7.937735	-7.953163	-7.958541	-7.958489
4.0	-7.935825	-7.950753	-7.956685	-7.956635
4.5	-7.933356	-7.947614	-7.954316	-7.954274

Table S3: CCSDT potential energy curve of the LiH dissociation for different basis sets.

	cc-pvdz(conv)	cc-pvtz(conv)	cc-pvdz(TC)	cc-pvdz(TC-n)
1.0	-7.921428	-7.941695	-7.949142	-7.949140
1.2	-7.984112	-8.005593	-8.013040	-8.013036
1.4	-8.008435	-8.030931	-8.037609	-8.037603
1.6	-8.014803	-8.036709	-8.042665	-8.042657
1.8	-8.011708	-8.032858	-8.037787	-8.037777
2.0	-8.003855	-8.024491	-8.028333	-8.028321
2.2	-7.993904	-8.014192	-8.017100	-8.017085
2.4	-7.983425	-8.003400	-8.005664	-8.005645
2.6	-7.973322	-7.992921	-7.994886	-7.994860
2.8	-7.964125	-7.983227	-7.985246	-7.985215
3.0	-7.956146	-7.974609	-7.977008	-7.976971
3.2	-7.949550	-7.967248	-7.970294	-7.970249
3.4	-7.944368	-7.961245	-7.965088	-7.965041
3.6	-7.940499	-7.956593	-7.961252	-7.961200
3.8	-7.937735	-7.953170	-7.958542	-7.958490
4.0	-7.935826	-7.950760	-7.956687	-7.956636
4.5	-7.933357	-7.947622	-7.954316	-7.954274

Table S4: CCSDTQ potential energy curve of the LiH dissociation for different basis sets.

2 Be_2 molecule

	CCSD	CCSD(T)	CCSDT	TC-CCSD-n	TC-CCSD(T)-n
1.6	-29.138617	-29.144799	-29.145515	-29.259292	-29.264955
1.8	-29.190615	-29.196132	-29.196938	-29.302146	-29.306777
2.0	-29.215711	-29.220591	-29.221498	-29.318618	-29.322443
2.2	-29.226706	-29.230867	-29.231803	-29.322227	-29.325283
2.454	-29.231412	-29.234614	-29.235463	-29.319984	-29.322166
2.6	-29.232234	-29.234920	-29.235675	-29.317973	-29.319743
2.8	-29.232702	-29.234774	-29.235384	-29.315667	-29.316989
3.2	-29.233431	-29.234629	-29.234985	-29.313462	-29.314199
3.6	-29.234264	-29.234957	-29.235151	-29.313228	-29.313635
4.0	-29.234853	-29.235272	-29.235375	-29.313493	-29.313715
4.4	-29.235132	-29.235402	-29.235458	-29.313687	-29.313807
4.8	-29.235211	-29.235400	-29.235432	-29.313750	-29.313814
5.2	-29.235203	-29.235346	-29.235365	-29.313742	-29.313774
5.6	-29.235171	-29.235288	-29.235300	-29.313711	-29.313724
8.0	-29.235077	-29.235156	-29.235158	-29.313606	-29.313592

Table S5: Potential energy curve of the Be_2 dissociation in the cc-pVDZ basis.

	TC-CCSDT-n	TC-CCSD	TC-CCSD(T)	TC-CCSDT
1.6	-29.266377	-29.259238	-29.264936	-29.266441
1.8	-29.308459	-29.302104	-29.306764	-29.308524
2.0	-29.324286	-29.318582	-29.322434	-29.324352
2.2	-29.327114	-29.322200	-29.325278	-29.327177
2.454	-29.323714	-29.319970	-29.322171	-29.323774
2.6	-29.321051	-29.317968	-29.319755	-29.321113
2.8	-29.317977	-29.315675	-29.317011	-29.318035
3.2	-29.314713	-29.313486	-29.314232	-29.314766
3.6	-29.313900	-29.313263	-29.313675	-29.313948
4.0	-29.313858	-29.313530	-29.313755	-29.313901
4.4	-29.313892	-29.313724	-29.313846	-29.313931
4.8	-29.313871	-29.313786	-29.313851	-29.313908
5.2	-29.313818	-29.313778	-29.313811	-29.313855
5.6	-29.313763	-29.313746	-29.313760	-29.313799
8.0	-29.313625	-29.313641	-29.313627	-29.313660

Table S6: Potential energy curve of the Be_2 dissociation in the cc-pVDZ basis.

	CCSD	CCSD(T)	CCSDT	TC-CCSD-n	TC-CCSD(T)-n
1.6	-29.208281	-29.215393	-29.216058	-29.265583	-29.270771
1.8	-29.259412	-29.265685	-29.266474	-29.312408	-29.316689
2.0	-29.284102	-29.289617	-29.290516	-29.332647	-29.336192
2.2	-29.294932	-29.299650	-29.300582	-29.339670	-29.342514
2.454	-29.299578	-29.303272	-29.304124	-29.340858	-29.342898
2.6	-29.300390	-29.303542	-29.304300	-29.340323	-29.341975
2.8	-29.300851	-29.303362	-29.303973	-29.339525	-29.340745
3.2	-29.301568	-29.303177	-29.303542	-29.339021	-29.339659
3.6	-29.302391	-29.303489	-29.303693	-29.339506	-29.339806
4.0	-29.302974	-29.303796	-29.303911	-29.340063	-29.340174
4.4	-29.303246	-29.303921	-29.303990	-29.340376	-29.340382
4.8	-29.303323	-29.303917	-29.303960	-29.340489	-29.340438
5.2	-29.303310	-29.303858	-29.303891	-29.340503	-29.340418
5.6	-29.303275	-29.303797	-29.303823	-29.340481	-29.340376
8.0	-29.303176	-29.303662	-29.303678	-29.340380	-29.340247

Table S7: Potential energy curve of the Be_2 dissociation in the cc-pCVDZ basis.

	TC-CCSDT-n	TC-CCSD	TC-CCSD(T)
1.6	-29.272850	-29.265549	-29.270781
1.8	-29.318934	-29.312382	-29.316698
2.0	-29.338480	-29.332628	-29.336201
2.2	-29.344662	-29.339657	-29.342526
2.454	-29.344638	-29.340858	-29.342917
2.6	-29.343430	-29.340331	-29.342000
2.8	-29.341832	-29.339546	-29.340779
3.2	-29.340234	-29.339059	-29.339705
3.6	-29.340122	-29.339552	-29.339858
4.0	-29.340363	-29.340110	-29.340225
4.4	-29.340512	-29.340423	-29.340432
4.8	-29.340539	-29.340536	-29.340486
5.2	-29.340508	-29.340549	-29.340465
5.6	-29.340460	-29.340526	-29.340423
8.0	-29.340327	-29.340426	-29.340294

Table S8: Potential energy curve of the Be_2 dissociation in the cc-pCVDZ basis.

	CCSD	CCSD(T)	CCSDT
1.6	-29.167245	-29.176188	-29.176690
1.8	-29.215982	-29.223684	-29.224312
2.0	-29.238509	-29.245192	-29.245952
2.2	-29.247137	-29.252821	-29.253660
2.454	-29.249009	-29.253463	-29.254273
2.6	-29.248585	-29.252396	-29.253137
2.8	-29.247865	-29.250915	-29.251536
3.2	-29.247269	-29.249227	-29.249618
3.6	-29.247380	-29.248687	-29.248917
4.0	-29.247561	-29.248495	-29.248628
4.4	-29.247637	-29.248362	-29.248444
4.8	-29.247614	-29.248227	-29.248282
5.2	-29.247539	-29.248090	-29.248131
5.6	-29.247449	-29.247965	-29.247999
8.0	-29.247182	-29.247649	-29.247671

Table S9: Potential energy curve of the Be_2 dissociation in the cc-pVTZ basis.

	TC-CCSD-n	TC-CCSD(T)-n	TC-CCSDT-n
1.6	-29.250927	-29.259377	-29.259973
1.8	-29.296447	-29.302984	-29.304222
2.0	-29.315255	-29.320473	-29.322089
2.2	-29.320435	-29.324550	-29.326296
2.454	-29.320061	-29.323011	-29.324593
2.6	-29.319239	-29.321653	-29.323037
2.8	-29.318270	-29.320106	-29.321197
3.2	-29.317229	-29.318314	-29.318946
3.6	-29.317074	-29.317735	-29.318104
4.0	-29.317229	-29.317648	-29.317875
4.4	-29.317389	-29.317674	-29.317828
4.8	-29.317472	-29.317683	-29.317805
5.2	-29.317497	-29.317667	-29.317771
5.6	-29.317490	-29.317637	-29.317733
8.0	-29.317404	-29.317516	-29.317604

Table S10: Potential energy curve of the Be_2 dissociation in the cc-pVTZ basis.

References

[1] Szenes, K.; Mörchen, M.; Fischill, P.; Reiher, M. Striking the Right Balance of Encoding Electron Correlation in the Hamiltonian and Wavefunction Ansatz. *Faraday Discuss.* **2024**, *254*, 359–381.
